# The Role of the Multidisciplinary Evaluation of Interstitial Lung Diseases: Systematic Literature Review of the Current Evidence and Future Perspectives

**DOI:** 10.3389/fmed.2019.00246

**Published:** 2019-10-31

**Authors:** Federica Furini, Aldo Carnevale, Gian Luca Casoni, Giulio Guerrini, Lorenzo Cavagna, Marcello Govoni, Carlo Alberto Sciré

**Affiliations:** ^1^Section of Rheumatology, Department of Medical Sciences, University of Ferrara and Azienda Ospedaliero-Universitaria Sant'Anna di Ferrara, Cona, Italy; ^2^Department of Radiology, Azienda Ospedaliero-Universitaria Sant'Anna di Ferrara, Cona, Italy; ^3^Department of Medical Sciences, Research Centre on Asthma and COPD, University of Ferrara, Ferrara, Italy; ^4^Division of Rheumatology, University and IRCCS Policlinico S. Matteo Foundation, Pavia, Italy

**Keywords:** interstitial lung disease (ILD), connective tissue disease (CTD), multidisciplinary team (MDT), rheumatologist, interstitial pneumonia with autoimmune features (IPAF)

## Abstract

The opportunity of a multidisciplinary evaluation for the diagnosis of interstitial pneumonias highlighted a major change in the diagnostic approach to diffuse lung disease. The new American Thoracic Society, European Respiratory Society, Japanese Respiratory Society, and Latin American Thoracic Society guidelines for the diagnosis of idiopathic pulmonary fibrosis have reinforced this assumption and have underlined that the exclusion of connective tissue disease related lung involvement is mandatory, with obvious clinical and therapeutic impact. The multidisciplinary team discussion consists in a moment of interaction among the radiologist, pathologist and pulmonologist, also including the rheumatologist when considered necessary, to improve diagnostic agreement and optimize the definition of those cases in which pulmonary involvement may represent the first or prominent manifestation of an autoimmune systemic disease. Moreover, the proposal of classification criteria for interstitial lung disease with autoimmune features (IPAF) represents an effort to define lung involvement in clinically undefined autoimmune conditions. The complexity of autoimmune diseases, and in particular the lack of classification criteria defined for pathologies such as anti-synthetase syndrome, makes the involvement of the rheumatologist essential for the correct interpretation of the autoimmune element and for the application of classification criteria, that could replace clinical pictures initially interpreted as IPAF in defined autoimmune disease, minimizing the risk of misdiagnosis. The aim of this review was to evaluate the available evidence about the efficiency and efficacy of different multidisciplinary team approaches, in order to standardize the professional figures and the core set procedures that should be necessary for a correct approach in diagnosing patients with interstitial lung disease.

## Introduction

Multidisciplinary discussion (MDD) is currently recommended during the diagnostic process of interstitial lung diseases (ILD) in particular when idiopathic pulmonary fibrosis (IPF) is suspected (1, 2). IPF has the worst prognosis among the different forms of ILD, with a median survival of 3–5 years from the diagnosis. It can generally be suspected in male subjects over the age of 60 who present an usual interstitial pneumonia pattern (UIP) at radiology and histology. In subjects with a radiological pattern compatible with UIP and in the absence of a detectable etiology, surgical lung biopsy (SLB) is not necessary, whereas it should be considered in patients with probable or indeterminate radiological patterns for UIP especially when an alternative diagnosis is not achievable (1). MDD is currently replacing the histological evaluation, due to its limited reliability and intrinsic risks particularly in elderly or highly comorbid patients (3). Given the poor prognosis of IPF and the availability of new anti-fibrotic drugs such as pirfenidone and nintedanib, the diagnosis formulated via MDD is currently considered the gold standard (4–6). Despite this guideline for IPF diagnosis, there are no available studies that clearly assess the impact of multidisciplinary team (MDT) in the approach to patients with ILD and we do not know if the evaluation by experts can actually be better than MDD. Nonetheless, the participation by clinicians, radiologists, and when applicable histopathologists, could be considered useful to share clinical cases between physicians with different points of view in order to establish a “common language” and improve the knowledge of the singles (7).

Applying the guidelines of the American Thoracic Society, European Respiratory Society, Japanese Respiratory Society and Latin American Thoracic Association (ATS/ERS/JRS/ALAT), the recommended MDT is generally composed by a clinician (often a pulmonologist), a thoracic radiologist and pathologist with experience in ILD. Other physicians as rheumatologist should be considered only in selected cases (8). Current clinical practice guidelines for IPF recommend to perform a battery of serological test as C-reactive protein (CRP), erythrocyte sedimentation rate (ESR), antinuclear antibodies (ANA) by immunofluorescence, rheumatoid factor (RF), myositis panel, and anti–cyclic citrullinated peptide (ACPA) without a previous consultation with rheumatologist, reserving this possibility in case of positivity of serological tests or presence of clinical manifestations suggesting an underling rheumatological disease (especially in women <60 years old) (8).

Hence ILD could be related to rheumatoid arthritis (RA), systemic vasculitis (especially antineutrophil Cytoplasmic Antibodies (ANCA)-associated Vasculitis) (9) and different connective tissue disease (CTD) especially systemic sclerosis (SSc), myositis spectrum disorders comprising overlap myositis and antisynthetase syndrome (ASSD) but also systemic lupus erythematosus, primary Sjoġren's syndrome, and mixed CTD (10, 11). Specific classification criteria are available for most CTDs, while classification criteria currently lack for diseases such as ASSD, making the correct diagnosis very challenging (12).

The recent introduction of criteria defining interstitial pneumonias with autoimmune features (IPAF) has allowed to reclassify those ILD that did not meet any CTD criteria, creating a growing interest in research concerning these new entities, especially on their possible evolution in CTD and overall prognosis (13).

The primary objective of this study was to perform a systematic review of literature to explore the evidence on the organization and outcome of MDT for the diagnosis and management of ILD, and to evaluate the role of rheumatologist. A secondary objective is to elaborate a definite proposal of ILD multidisciplinary evaluation.

## Materials and Methods

A systematic literature review was performed using electronic databases Pubmed (1999–2019) and Embase (1999–2019). The search strategy was elaborated to include the greatest number of references dealing with the populations and the interventions object of the study by using the following keywords in combination with the Boolean operators OR and AND: “*interstitial,” “pneumonia,” “multidisciplinary,” “lung disease, interstitial,” “pulmonary fibrosis,” “interstitial pneumonias,” “multidisciplinary team,”* and “*multidisciplinary approach.”* Three reviewers (FF, GG, and AC) independently screened the titles and abstracts of all retrieved papers and selected the studies to be included in this review, after removing duplicates. All the articles selected by at least one of the reviewers were retrieved for full text evaluation. Article were selected according *a priori* inclusion criteria according to PICO methodology: (a) population: subjects aged>18 years with a suspected or established diagnosis of ILD; (b) intervention: multidisciplinary approach involving at least two different physicians of two different specialties; (c) type of study: metanalysis, randomized controlled trial (RCT), cohort, case control and case series (>5 patients) in English language. Other languages and other study designs (narrative review, case reports and meeting abstracts) were excluded. In case of disagreement between the reviewers, a further author (CS) was consulted to achieve a consensus. Primary outcome of this systematic review was the definition of the organization and physicians involved in the MDT with particular attention to clinical data collected and instrumental exams performed. A secondary objective was to evaluate the outcome of multidisciplinary approach (e.g., diagnosis or management) and to evaluate the role of rheumatologist. Selected articles were reviewed independently by three reviewers (FF, GG, and AC) and all data were extracted using an extraction form designed to respond to primary and secondary objectives of the review. The following data were extracted: authors, journal, year of publication, study design, inclusion and exclusion criteria, number of participants, population (ILD onset or established ILD, IPF, CTD related ILD, or both), interventions (physicians involved, instrumental examinations considered during the MDD) and outcomes evaluated (diagnosis, prognosis, efficacy of a treatment and other).

## Results

**Figure 1:**
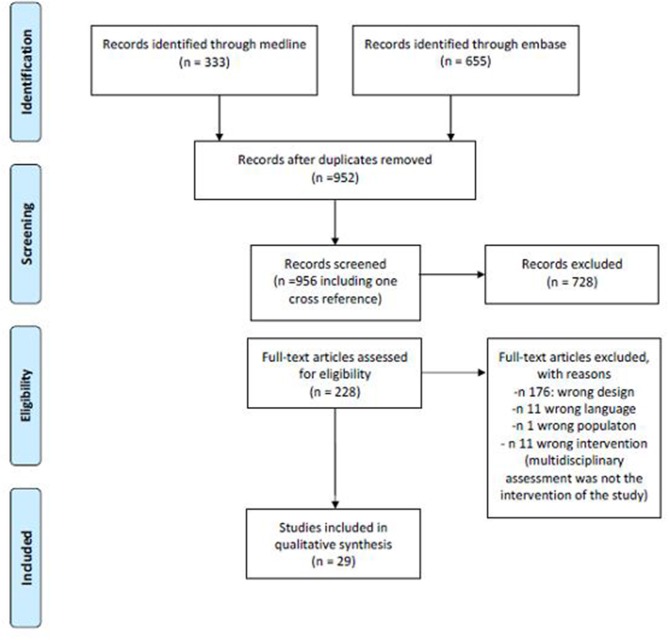
Literature search flow chart.

The search provided a total number of 333 citations from Pubmed and 955 from Embase. After excluding duplicates, a total of 952 references were screened for title and abstract and a total of 228 (including one cross reference) for full text analysis. A total of 29 papers were finally included for data extraction. Figure 1 summarizes the number of papers excluded and the reason for exclusion. Table 1 summarizes the main characteristics of the included studies.

**Table 1:** Characteristics and results of selected studies.

**References**	**Study design**	**Population**	**Number of participants**	**Mean age(years) mean ± SD or (IQR)**	**Female %**	**Mean follow-up (months)**
Burge et al. (14)	Retrospective cohort	ILD onset	71	/	/	/
Chartrand et al. (15)	Retrospective cohort	ILD established, myositis spectrum of disease, and/or SynS	33	55	22 (66.7%)	/
Castelino et al. (16)	Retrospective cohort	ILD onset	50	64 (32–80)	27 (54%)	12
De Sadeleer et al. (17)	Retrospective cohort	ILD onset	938	60.8 (14–90)	34.8%	
Ferri et al. (18)	Retrospective case-control	UCTD, IPAF, U-ILD	52 UCTD vs. 50 (35 IPAF- 15 U-ILD)	UCTD 55 ± 13, IPAF 63 ± 12, U-ILD 68 ± 8.9	UCTD 44 (86%) IPAF 24(69%) U-ILD 9(60%)	/
Flaherty et al. (19)	Retrospective cohort	ILD onset (CTD excluded)	58	/	/	/
Fujisawa et al. (20)	Retrospective cohort	ILD onset (subjected to Surgical Lung Biopsy)	465	65	35%	7
Han et al. (21)	Retrospective cohort	Idiopathic ILD	56	56.9 ± 12.6	32 (57.1%)	7
Jeong et al. (22)	Prospective cohort	ILD related to CTD Idiopathic ILD	44 (23 CTD-ILD vs. 21 IPF)	CTD-ILD: 58.5, Idiopathic ILD: 70	CTD-ILD: 69.6%, Idiopathic ILD: 23.8%	
Jo et al. (23)	Retrospective cohort	Idiopathic ILD	417		31	26.16
Jo et al. (24)	Retrospective cohort	Idiopathic ILD, ILD related to CTD, unclassifiable ILD	90	67 ± 11	36 (40%)	/
Kalluri et al. (25)	Retrospective case-control/retrospective cohort	Idiopathic ILD	32	MDC group: 22, no MDC group: 10	MDC group: 36%, no MDC group: 40%	No MDC group: 17.4; MDC group: 14.4
Kohashi et al. (26)	Retrospective cohort	Idiopathic ILD that underwent to SLB	47	62 (56–67)	14 (29.8%)	1,582 (1,213–1,935) days
Kondoh et al. (27)	Retrospective cohort	Idiopathic ILD, Unclassifiable ILD, NSIP, hypersensitivity Pneumonia, ILD related to CTD	179	65 (60–70)	56 (31.3%)	/
Levi et al. (28)	Prospective cohort	New onset: ILD related CTD, Idiopathic ILD, IPAF	60	67.3 ± 12	27(45%)	/
Lok (29)	Retrospective cohort	/	138	General respiratory clinic: 64.6 vs. Patients of ILD clinic: 55.9	General respiratory clinic 31 (37%) ILD clinic: 28 (52%)	General respiratory clinic 31.9 vs. ILD clinic 22.3
Chaudhuri et al. (30)	Retrospective cohort	ILD established: ILD related to CTD, Idiopathic ILD	318	/	/	/
Nakamura et al. (31)	Retrospective cohort	U-ILD	33	64.4 ± 8.8	17(51.5%)	60.5
Newton et al. (32)	Retrospective cohort	familial pulmonary fibrosis	115	58 ± 10	57 (49.6%)	180
Patterson et al. (3)	Case control study	ILD onset	327 (80 of age>70)	54 ± 12 non-elderly vs. 76 ± 4 elderly	115(47%) non-elderly, 54 (68%) elderly	/
Pezzuto et al. (33)	Retrospective cohort	ILD onset	124	69 ± 7.9	37 (29.8%)	/
Tanizawa et al. (34)	Retrospective cohort	ILD established (UIP pattern at histology) CTD-ILD related are excluded	252.215 IPF, 19 U-ILD, 13 hypersensitivity pneumonitis	68.1 (62.1–72.6) with BCF vs. 67.7 (62.5–73.8) without BCF	32 (33.3%) in with BCF vs. 43(27.6%) without BCF	/
Thomeer et al. (35)	RCT	ILD established	182	18–75	NA	12
Tomassetti et al. (36)	Cross sectional	ILD established (without define UIP pattern on HRCT)	117 (59 BLC vs. 59 SLB)	59 (29–77) BLC vs. 59 (34–74) SLB	31 (53.4%) in BLC vs. 31 (52.5%) in SLB	
Tominaga et al. (37)	Retrospective cohort	Idiopathic ILD	95	63 (40–79)	17 (10.7%)	/
Oltmanns et al. (38)	Retrospective cohort	ILD established	63	68 ± 7	16. (25%)	11 ± 7
Ussavarungsi et al. (39)	Retrospective cohort	U-ILD	74	63 (20–89)	33(45%)	/
Walsh et al. (40)	Retrospective cohort	ILD onset	70	60.9 ± 15.5	46(66%)	67
Yamauchi et al. (41)	Prospective cohort	Idiopathic ILD	30	64.5 ± 6.3	8(26.7%)	/

*SD, standard deviation; IQR, interquartile range; MDT, multidisciplinary team; ILD, interstitial lung disease; HRCT, high resolution computer tomography; PFT, pulmonary function test; CT, computed tomography; ASSD/Syns, antisynthetase; UCTD, undifferentiated connective tissue disease; IPAF, Interstitial pneumonia with autoimmune features; U-ILD, undifferentiated connective tissue disease; CTD, connective tissue diseases; CTD-ILD, interstitial lung disease related to connective tissue diseases; MDC, multidisciplinary collaborative; SLB, surgical lung biopsy; NSIP, nonspecific interstitial pneumonia; UIP, usual interstitial pneumonia; BLC, bronchoscopic lung biopsy; /, not reported*.

### Physician Involved in the MDT

In the included studies, the professional figures most frequently involved in MDT were: pulmonologist (29/29), thoracic radiologist (26/29), and thoracic pathologist (23/29). The rheumatologist role was described in 7 studies. Other professional figures were reported in 7 studies, including: clinical nurse specialist, cardiothoracic surgeon and lung transplantation team, occupational therapists, cardiologist, immunologist, palliative care expert, respiratory therapist, physiotherapist, and dietitian.

Some studies compared different compositions of MDT. Lok performed a comparison between a general respiratory clinic composed only by a pneumologist and a nurse (84 patients) and an ILD clinic setting including a specialist with interest in ILD with the support of radiologist, pathologist, and access to transplant and cardiothoracic program (54 patients). A multidisciplinary approach-based follow-up seemed to give an advantage in terms of survival in patients aged <60 years, being age an important negative prognostic factors in this population (29). In the study by Burge et al., the MDT was composed by a clinical nurse specialist as well as the classical organization which included specialist radiologist, histopathologist, and clinician. The authors highlighted the importance of MDD in the diagnosis of ILD compared to histology. The 71 patients in the study had in fact undergone video-assisted thoracoscopic surgery (VATS), and a retrospective analysis by MDT of the histological, clinical and radiological data was performed. In 30% of cases after MDD the diagnosis differed significantly from the histology report, and in a further 12% MDD changed the diagnosis from probable to confident (14).

Not all cases must necessarily be submitted to MDD. Chaudhuri et al. applied the MDD in the retrospective evaluation of 318 patients. The MDT of this study met weekly, and only patients sent by ILD expert clinicians were evaluated. The authors emphasized that after the multidisciplinary analysis the diagnosis could change, and that in doubtful cases, where biopsy was not possible due to comorbidities, the diagnosis could be reconsidered and reviewed over time based on the evolution and any response to therapy (30). Flaherty et al. highlighted how in the evaluation of patients with suspected IPF the review of the case by the radiologist, pathologist and clinician is fundamental, and that the sharing of clinical, radiological and possibly histopathological information can modify the diagnosis and/or increase diagnostics confidence and interobserver agreement. The diagnostic process described in this study was in fact organized through 4 different steps during which more information were progressively shared and the progressive interaction between the MDT members was permitted. The agreement between clinicians and radiologists was thus increased from the beginning to the end of the diagnostic process (0.39 vs. 0.88) (19).

The multidisciplinary approach, while representing the gold standard in the diagnosis of ILD, is not always practicable in normal clinical routine since local structures may not have experts in this field or meetings may be difficult to organize due to the geographical distance between the participants or time-related limits. A solution to overcome these limits could be provided by digital platforms. Fujisawa et al. validated a digital platform for the organization of MDD. The clinical data and radiological and histological images of 465 patients with suspected ILD (all therefore subjected to SLB) were included in an electronic database accessible via the web. Each patient was given a numerical identification code. The members of the MDT (clinicians, radiologists, and pathologists) could then separately access the various information and then a web conference to discuss with the other two members of the MDT. Also in this study, the MDD made possible to reformulate the initial diagnosis in a conspicuous number of cases (49%), and from the analysis of the survival curves it was shown that also this MDD modality is able to identify those diagnoses with the worse prognosis (like IPF) [(20); Table 2].

**Table 2:** Physicians involved in the MDT.

**References**	**Pulmonologist**	**Radiologist**	**Pathologist**	**Rheumatologist**
Burge et al. (14)	1	1	1	0
Chartrand et al. (15)	1	1	1	1
De Sadeleer et al. (17)	1	1	1	1
Ferri et al. (18)	1	1	1	1
Kondoh et al. (27)	1	1	1	0
Levi et al. (28)	1	1	1	1
Jo et al. (24) 10/1/2019 9:37:00 p.m.	1	1	1	1
Flaherty et al. (19)	1	1	1	0
Fujisawa et al. (20)	1	1	1	0
Han et al. (21)	1	1	1	0
Jo et al. (42)	1	1	1	0
Kohashi et al. (26)	1	1	1	0
Lok (29)	1	1	1	0
Chaudhuri et al. (30)	1	1	1	0
Nakamura et al. (31)	1	1	1	0
Patterson et al. (3)	1	1	1	0
Pezzuto et al. (33)	1	1	1	0
Tanizawa et al. (34)	1	1	1	0
Thomeer et al. (35)	1	1	1	0
Tomassetti et al. (36)	1	1	1	0
Tominaga et al. (37)	1	1	1	0
Oltmanns et al. (38)	1	1	1	0
Walsh et al. (40)	1	1	1	0
Yamauchi et al. (41)	1	1	1	0
Jeong et al. (22)	1	1	0	1
Newton et al. (32)	1	1	0	0
Ussavarungsi et al. (39)	1	1	0	0
Castelino et al. (16)	1	0	0	1
Kalluri et al. (25)	1	0	0	0

### Variables Evaluated During MDD

Clinical history assessment is reported in 24 of the 29 included studies. In addition to demographics (age and sex), the most frequently collected data concerned smoke (17/29) and environmental exposure (11/29). The evaluation of symptoms related to the possible presence of CTD and physical examination were reported in 7 studies.

High resolution computed tomography (HRCT) was evaluated in all studies except two: one dealing with a multidisciplinary approach not for the diagnosis, but for palliative care of ILD patients (25), and one focused on transbronchial lung cryobiopsy (39). HRCT was usually acquired only at baseline during the diagnostic process (24/27 studies). In 3 studies including longitudinal information, HRCT was repeated after 3–6 months in two studies (22, 31), and not specified in one study (35). Baseline chest X-ray was described in only one study (37).

Pulmonary function tests (PFT) were part of the core set of parameters analyzed during multidisciplinary evaluation in almost all studies (27/29). PFT were not performed in the same two previously described studies, in which even HRCT was not performed (25, 39). In 21 studies, PFTs were performed only at the baseline while in 6 studies repetition was described during follow-up with different timing: 1–3 months (38), 3 months (22), 3–6 months (31), annually (27), and not specified (32). The parameters considered were in most cases the forced vital capacity (FVC), the ability to spread carbon monoxide (DLCO) and forced expiratory volume in the 1st second (FEV1); less frequently, total lung capacity (TLC); and residual volume (RV).

Pulmonary histology was evaluated in 21 studies. The role in the MDD of biopsy and especially of two different techniques (namely surgical lung biopsy SLB, and bronchoscopic lung biopsy BLC) was evaluated in a cross-sectional study involving 171 patients (58 BLC vs. 59 SLB). Both the modalities of biopsy increased the diagnostic accuracy of IPF (36). Ussavarungsi evaluated the role of Transbronchial Cryobiopsy (TBC) in the MDD; in this series of 74 patients, TBC failed to obtain histological samples demonstrating a specific UIP or NSIP pattern (39). In a retrospective cohort of 124 patients with suspected IPF, authors suggested to perform HRCT at baseline together with PFT (FVC, TLC; RV and DLCO), laboratory test for CTD and vasculitis, and bronchoalveolar lavage (BAL) for cytological and microbiological tests. HRCT results were then reviewed by MDT and classified according to the ATS/ERS/JRS/ALAT guidelines in UIP pattern, probable UIP and inconsistent with UIP patterns. Only in the last two and in presence of clinical, immunological, microbiological, and cytological abnormalities suggestive for IPF, the authors recommended biopsy. 15/124 patients could not be classified in neither of proposed definitions of HRCT patterns, but they were subsequently diagnosed with IPF after MDD and biopsy (33).

Serological data were reported in 17/29 studies, and 14 included autoantibody profile tests, especially RF, ACPA, ANA, antibodies against extractable nuclear antigens (ENA), myositis specific antibodies (including anti-synthetase) and myositis associated. Two studies reported genetic evaluation. Newton correlated traditional parameters evaluated during MDD (demographic data, physical examination, PFT, and HRCT) with four telomere-related genes mutations (TERT, TERC, RTEL1, and PARN). These genetic investigations were not usually performed during the traditional MDD for ILD, but this study focused on the evaluation of hereditary forms of pulmonary fibrosis (32). Another genetic test relating the MUC5B gene (rs35705950), associated with susceptibility to IPF, was obtained in a study cohort involving 252 ILD patients considered through MDD for diagnosis. In this study, the presence of bronchiolocentric fibrosis seemed not to correlated with MUC5B gene, telomere length, and IPF diagnosis formulated through MDD (34).

Further instrumental investigations evaluated during MDD were described in 15 studies, including BAL, doppler echocardiography, and 6-min walking test (Table 3).

**Table 3:** Variables evaluated during MDD.

**References**	**Clinical evaluation**	**HRCT**	**PFT**	**Lung biopsy**	**Laboratory test**	**Other**
Burge et al. (14)	History (brief clinical history, the duration of breathlessness, exposure, and smoking histories) Physical examination (crackles and clubbing)	Yes, pre-operative lung CT	Full lung function tests before biopsy (not described)	Yes	Immunological tests to identify collagen-vascular diseases, antibodies associated with hypersensitivity pneumonitis, and angiotensin converting enzyme levels	/
Chartrand et al. (15)	History (smoke, family history) BMI	Yes at baseline	Yes, FVC, DLCO	No	5 myositis-specific (Jo1, PL12, PL7, OJ, EJ, Mi2, SRP) and myositis-associated antibodies (Ro52, Ku, PM-Scl) antibodies (Jo1, PL-7, PL-12, EJ, OJ), 2 other myositis-specific antibodies (Mi-2, SRP), and 3 myositis-associated antibodies (Ku, PM-Scl, Ro-52)	/
Castelino et al. (16)	History (occupational and environmental exposures, medication history, family history) Physical examination (skin, mucus membranes, musculoskeletal, oropharyngeal, and gastrointestinal system)	Yes at baseline	Yes, FVC, DLCO	Yes	Anti-nuclear antibody (performed using HEp2 cell lines at BWH), ENAs, RF, inflammatory markers (ESR and CRP)	-Nailfold capillaroscopy -Echocardiography -Esophageal testing for pH or manometric studies
De Sadeleer et al. (17)	History (familial history, exposures, comorbidities, and medication use) -Physical examination	Yes at baseline	Yes not specified	Yes	Serological data (not specified)	BAL
Ferri et al. (18)	- History (demographic, occupational, smoking, medication, environmental, occupational, autoimmune manifestation)	Yes at baseline	Yes, including DLCO	Surgical lung biopsy Skin biopsy	ANA, anti-ENA, ESR, CRP, routine blood chemistry, urinalysis, infections, RF (first line), antiCCP, complement, ASMA, AMA, ANCA, antiphospholipid, organ specific antibodies, 24 h proteinuria (second line)	Doppler echocardiography, Joint echography, Nailfold capillaroscopy, Schirmer's test, Salivary gland echography, Minor salivary gland biopsy, Muscle biopsy, Electromyography
Flaherty et al. (19)	History (symptoms, environmental exposures, comorbid illnesses, medication use, smoking history, family history) -Physical examination findings	Yes at baseline	Yes, lung volumes and DLCO	No	Serological data (not specified)	/
Fujisawa et al. (20)	History (symptoms, environmental exposures, smoking history, family history, comorbid illnesses) -Physical examination	Yes, within 3 months from SLB	Yes, FVC, FEV1, DLCO	Yes	Blood test results, arterial blood gas analysis (or SpO2)	6-MWT, bronchoscopy, including bronchoalveolar lavage
Han et al. (21)	- History [smoking history; environmental, occupational and drug exposure; history of established connective tissue disease (CTD)]	Yes at baseline	Yes not specified	Yes	No	/
Jeong et al. (22)	- History (exercise status, Educational status, underlying rheumatic diseases)	Yes, repeat at 6 months	Yes, lung volumes, and DLCO, repeat at 3 months	No	No	The Brief Illness Perception Questionnaire (IPQ), Beliefs about Medicines Questionnaire (BMQ), Patient Health Questionnaire-2 (PHQ-2), Adherence measures
Jo et al. (42)	History (smoke, presence of underlying rheumatic diseases) -Physical examination(BMI)	Yes at baseline	Yes, FVC, FEV1/FVC, and DLCO	Yes	No	/
Jo et al. (24)	-History smokers (pack/years)	Yes at baseline	Yes, FVC, TLC, DLCO	Yes	Extended myositis screen and hypersensitivity precipitins and BNP	6-MWT, Resting SpO2, Nadir SpO2, Transthoracic echocardiogram, right heart catheterization
Kalluri et al. (25)	-Charlson Comorbidity Index -Pharmacotherapy (anti fibrotics, PPI, opioids, benzodiazepines)	No	Yes, FVC, DLCO	No	No	/
Kohashi et al. (26)	-History (smoke) - BMI	Yes at baseline	Yes, FVC, FEV1, FEV1/FVC, DLCO	No	BNP, LDH, KL-6, SP-D, ANA, RF, other autoantibodies	echocardiography
Kondoh et al. (27)	-History (smoke)	Yes at baseline	Yes, FVC, DLCO, FEV1/FVC repeated every year	Yes	No	BAL, PaO2
Levi et al. (28)	-History (smoke, family history of ILD, medications and environmental risk factors)	Yes at baseline	Yes, FVC%, DLCO%, and TLC%	Yes	Complete blood count, chemistry, renal and liver function tests, antinuclear antibody, rheumatoid factor (RF), C-reactive protein (CRP), anti-dsDNA, Scl70, anti-SSA, and anti-SSB were done. A cyclic citrullinated peptide (CCP) antibodies test was done in the case of a positive RF result, anti-Jo1, anti-RNP, anti-Smith, anticentromere, antimyeloperoxidase, antiproteinase−3, and anticardiolipin antibodies, erythrocyte sedimentation rate, various IgG subclasses including IgG4, and vitamin D (level)	Echocardiogram (Pulmonary hypertension, right heart failure), O2 saturation, Bronchoscopy (BAL only, TBB, Cryobiopsy, EBUS), 6-min walking distance (6MWD) test,
Lok (29)	-Evaluation of ongoing pharmacologic therapy	Yes at baseline	Yes, FEV1,FVC,TLC, DLCO	Yes	No	/
Chaudhuri et al. (30)	No	Yes at baseline	Yes, lung volumes, and DLCO	No	No	/
Nakamura et al. (31)	-Evaluation of Smoking index -GAP (Gender, Age, and Physiology) score	Yes, every 3–6 months	Yes, FVC, FEV1, DLCO, DLCO/VA every 3–6 months	Yes	Krebs von der Lungen-6, surfactant protein D, antinuclear antibody, auto-antibodies related to connective tissue diseases	Echocardiography
Newton et al. (32)	History (ethnicity, clinical manifestations: dyspnea, cough, smoking status) -Physical examination (crackles, clubbing)	Yes at baseline	Yes, FVC DLCO at baseline and during follow up without a established timing	No	No	/
Patterson et al. (3)	-History (race, smoking habits, clinical features of sarcoidosis, hypersensitivity pneumonitis, and CTD related ILD)	Yes at baseline	Yes, FVC, and DLCO at baseline and yearly	Yes	No	Walking distance, Hypoxemia
Pezzuto et al. (33)	No	Yes at baseline	Yes, at the time of evaluation FVC, TV, TLC, DLCO	Yes	For exclusion of CTD and vasculitis but not specified	BAL
Tanizawa et al. (34)	-History (ethnicity/race, smoking status, selected comorbidities) (asthma; congestive heart failure; gastroesophageal reflux; sleep apnea; diabetes), exposure history	Yes closed to biopsy. Categorized as definite UIP, possible UIP, or inconsistent with UIP pattern	Yes, close to biopsy FVC, FEV1, TLC, DLCO	Yes	No	MUC5B genotyping and telomere length measurement
Thomeer et al. (35)	No	Yes within 12 months before biopsy and during follow up	No	Yes	No	/
Tomassetti et al. (36)	-History: onset, symptoms, detailed history of exposure, family history, past medical history, and medications	Yes at baseline	Yes, at the time of evaluation FVC, RV, TLC, DLCO	No	Blood cell count, LDH, CRP, ESR, liver and kidney function profile, autoimmunity—ANA ENA ANCA	/
Tominaga et al. (37)	-History: onset, symptoms, detailed history of exposure, family history, past medical history, and medications	Yes, baseline	Yes VC, DLCO	Yes	Rheumatoid arthritis test, rheumatoid arthritis particle agglutination (RAPA) and ANA, serum biomarkers (Krebs von der Lungen-6 and surfactant protein-D)	/
Oltmanns et al. (38)	-History (comorbidities, smoking history)	Yes at baseline	/	Yes	Blood gas analysis, liver function test	/
Ussavarungsi et al. (39)	No	No	/	Yes	No	/
Walsh et al. (40)	-History (smoking habits, rheumatological disease, and rheumatological manifestation)	Yes at baseline	/	Yes	Autoantibodies	/
Yamauchi et al. (41)	-History (smoke)	Yes at baseline	/	No	KL-6, SP-D	/

*CT, computer tomography; BMI, body mass index; FVC, forced vital capacity; DLCO, the ability to spread carbon monoxide; FEV1, forced expiratory volume in the 1st second; less frequently, TLC, total lung capacity; CRP, C-reactive protein; ESR, erythrocyte sedimentation rate; ANA, antinuclear antibodies; RF, rheumatoid factor; ACPA, anti-cyclic citrullinated peptide; ENA antibodies against extractable nuclear antigens; BAL, bronchoalveolar lavage; ASMA, antibodies against smooth muscle; ANCAs, anti-neutrophil cytoplasmic antibodies; CTD, connective tissue disease; SLB, surgical lung biopsy; 6mwt, six minute walking test; ILD-CTD, interstitial lung disease related to connective tissue disease; IPF, idiopathic pulmonary fibrosis; SpO2, saturation of peripheral oxygene; BNP, natriuretic peptide B; LDH, lactic dehydrogenase; KL-6, Krebs von den Lungen 6; SP-D, surfactant protein-D; NSIP, idiopathic non-specific interstitial pneumonia; IPAF, interstitial pneumonia with autoimmune features; TBB, transbronchial biopsy; Scl70, anti-topoisomerase1; EBUS, endobronchial ultrasound; PaO2, Partial Pressure of Oxygen in Arterial Blood; U-ILD, undifferentiated interstitial lung disease; BCF, bronchiolocentric fibrosis; MUC5B, mucin 5B; /, not reported*.

### Outcome Evaluated by MDT

Fifteen studies had as outcome a reference standard diagnosis, 7 prognostic evaluation, 5 both diagnosis and prognosis, 1 evaluated efficacy of pirfenidone treatment, and 1 the effect of multidisciplinary approach on patient perception of the disease.

Evaluating in detail the studies in which the outcome was the diagnosis, after the assessment by the MDT of a large cohort of 417 patients collected in the Australian IPF Registry (AIPFR), it was shown that in 23% of cases the guidelines for IPF were not applied by referring physicians (42). Despite this observation, in another study by the same authors the MDD showed to be relevant not only for the diagnosis, but also for the investigations prescribed and therapeutic behavior. After multidisciplinary evaluation of 93 patients, in fact, ILD diagnosis was changed in 53% of patients referred, and 71% of unclassifiable disease were re-classified under a specific diagnosis with obvious implication on therapeutic approach including an increased recommendation for anti-fibrotic therapy and referral for clinical trials (24). In a larger study by De Sadeleer involving 938 patients sent for multidisciplinary evaluation, the diagnosis was reached in 79.5% and modified in 41.9% of cases after MDD, while a diagnostic conclusion was not achieved only in 19.5% of the patients; however, in this case further investigations (16% of the total court) were at least suggested. This study demonstrated that a correct diagnosis also correlated with better prognosis, and that MDT could be helpful for the identification of those patients with worse prognosis. Indeed patients who were diagnosed as IPF demonstrated a worse prognosis than those classified as not-IPF after MDD [Hazard ratio (HR) 4.31, *p* < 0.001], while patients initially classified as IPF who reported a change in their diagnosis after MDD showed a better prognosis compared to patients definitely diagnosed with IPF (HR 0.37, *p* = 0.094) (17). In another study of 33 patients with previous diagnosis of unclassifiable-ILD (U-ILD), clinical, radiological and histological data were retrospectively evaluated by MDT. After MDD, the initial diagnosis was confirmed in 18 (54.5%) patients, but changed to collagen vascular disease-related interstitial pneumonia in 9 (27.3%), to chronic hypersensitivity pneumonitis in 3 (9.1%), to idiopathic pleuro-parenchymal fibroelastosis in 2 (6.1%), and IPF with emphysema in 1 (3.0%) patient (31).

The importance of cooperation between clinicians, radiologists and pathologists was reinforced by the analysis of patients enrolled in the IFIGENIA trial, a randomized placebo-controlled trial conducted on patients with IPF in which N-Acetylcysteine was associated referred to standard therapy (azathioprine plus steroid). Patients diagnosed as IPF by the clinician were subjected to a commission of thoracic radiology experts who evaluated chest HRCT images and by expert pathologists who evaluated the results of biopsies if performed. The diagnosis of IPF was rejected in 12.8% of cases formulated by the expert clinician after reviewing the histology and HRCT images thus demonstrating the importance of the multidisciplinary collaboration between clinicians, expert radiologists, and pathologists for a correct diagnosis of IPF (35). The reliability of MDD composed by these professional figures was also assessed. Seven different MDTs assessed 70 cases, for a total of 490 diagnoses [CTD-related ILD (*n* = 146), IPF (*n* = 88), idiopathic NSIP (*n* = 50), hypersensitivity pneumonitis (*n* = 46), and others (*n* = 160)]. Inter-MDT agreement for a first-choice diagnosis of IPF was good (κ = 0.60), good for CTD-related ILD (κ = 0.64), but fair for idiopathic NSIP (κ = 0.25), and hypersensitivity pneumonitis (κ = 0.24). The authors therefore recognized the excellent performance of the MDT in diagnosing IPF for which better defined classification criteria are available than for other conditions, i.e., hypersensitivity pneumonitis. Furthermore, the highest frequency of CTD-ILD, demonstrated the importance of including a rheumatologist in the multidisciplinary evaluation of ILD (40).

Besides the diagnostic process, MDD could be performed to evaluate the prognosis of particular populations of ILD patients. In a prospective cohort study involving 327 subjects, multidisciplinary approach was employed to evaluate the role of age onset to determine both diagnosis and prognosis of ILD patients (3). MDT can also be used not only in the diagnosis of IPF but also to identify sub-populations of patients with a worse prognosis. In a study conducted on 47 patients with IPF confirmed after SLB and MDD, the multidisciplinary evaluation allowed to identify the presence of emphysema and its extent as negative prognostic factors for survival (26). In the evaluation of the patient's suitability for starting pirfenidone therapy, the multidisciplinary meeting, where clinicians, radiologists and pathologists discussed clinical and instrumental data, was essential to identify IPF patients (38).

Possible applications of MDD could encompass the management of ILD patients. In a study by Kalluri, subjects with ILD secondary to rheumatic diseases referred to the MDT (composed of pneumologist and rheumatologist), were compared with patients suffering from IPF followed according to a normal care setting. While the disease progression assessed through the worsening of the HRCT and PFT parameters was comparable, patients evaluated by MDD experienced greater satisfaction and more participation in their care path (22). A multidisciplinary approach in palliative care involving the participation of ILD experts, a palliative respiratory care expert, nurse, respiratory therapist, physiotherapist, and a dietitian, compared to the standard approach (namely ILD experts and a nurse) proved efficacy in improving the management of a small series of 32 patient, in terms of reduced number of emergency visits and hospital admissions (25).

There is little evidence concerning the role of MDT activity in the follow up. The diagnosis of ILD can change over time in light of new clinical or serological elements that may emerge in the course of the disease, as well as the progress and response to therapy. In a retrospective study of 56 patients evaluated during a 7-month average follow-up, it was shown how the re-evaluation of new clinical elements and a second HRCT by the pulmonologist and radiologist can modify the diagnosis of a first multidisciplinary discussion (10.7%), as well as the level of agreement (25% of cases). The multidisciplinary evaluation should therefore be a dynamic process not limited to the initial phase of the diagnostic process but also considered during the follow up (21). In a retrospective cohort study, 30 patients with a probable UIP pattern on HRCT and histology compatible or probable for UIP were identified by MDD. The evolution of the radiological data and the prognostic implications of patients who evolved radiologically were therefore evaluated against a specific HRCT pattern. In this case, the MDT and in particular the interaction between the radiologist and pathologist was fundamental to identify the target population of this study (41).

### Role of Rheumatologist

The rheumatologist was included in MDT in 7 studies. The retrospective study by Chartrand highlighted the role of the rheumatologist in the MDT while evaluating patients with ILD. From the National Jewish Health Metical database, the authors identified patients initially referred as IPF. After the multidisciplinary evaluation, the diagnosis was modified in 33 patients in ASSD (27/33) or a myositis spectrum disease (6/33). In these patients the identification of specific myositis antibodies (in particular anti-synthetase) or myositis associated were fundamental. The authors underlined that about a third of the patients was ANA negative, and so the research of the autoimmune profile should be extended to these antibodies that often recognize cytoplasmic antigens. Moreover, in 85% of cases at least one manifestation attributable to CTD was present, such as Raynaud's phenomenon, mechanic's hand, Gottron's papules, capillaroscopic alterations. Among these, the muscular manifestations were present only in a third of patients (15). A retrospective observational study of 50 patients, the MDD led to a final diagnosis of CTD-ILD in 25 patients, IPF in 15 and other forms of ILD in 10. In particular, in 7 of the 25 patients with CTD-ILD the pre-MDD diagnosis was IPF with completely different prognostic and therapeutic implications. Therapy therefore changed in 20 of 25 patients with CTD-ILD and in 4 of 15 patients with IPF after MDT evaluation (16). In the study by Ferri et al., the MDD was performed by a rheumatologist and a pneumologist. Other professional figures such as the thoracic radiologist, surgeon and pathologist were considered only in selected cases. Given the type of setting, the authors described a more detailed clinical and laboratory assessment set with particular attention to the evaluation of autoimmune clinical manifestations and serological investigations. In the evaluation of the patient, specific instrumental investigations were also included, such as nailfold capillaroscopy, joint and salivary glands ultrasound, suggesting an application based on clinical suspicion (18). In a prospective study of 60 patients the role of the rheumatologist in the classification of patients with ILD at the onset is again emphasized. The diagnostic process was divided into three phases: a first phase in which the traditional MDT was involved, consisting of pulmonologist, radiologist and pathologist, and a second one where a rheumatologist evaluated the cases independently. In the course of traditional MDD clinical information, PFT, HRCT, biopsy, and BAL when available were evaluated. Serological investigations routinely performed included ANA, anti-dsDNA, anti-topoisomerase-1(Scl70), anti-SSA, and anti-SSB, ACPA (done in the case of a positive RF result). To these tests, the following could be added after the rheumatologic evaluation: anti-Jo1, anti-RNP, anti-Smith, anticentromere, ANCA, and anticardiolipin antibodies, various IgG subclasses including IgG4. Also anti-synthetase antibodies were tested if deemed necessary by the rheumatologist. Finally, there was a third phase of comparison between MDT and rheumatologist, in which some diagnoses formulated by the MDT were modified. In particular 21.9% of IPF cases and 28.5% of hypersensitivity pneumonia cases (HP) the diagnosis was modified in favor of pathologies of rheumatological interest such as Sjogren's syndrome, associated ANCA-associated vasculitis, RA, ASSD, SSc, and related IgG4 pathology. The authors also argued that the rheumatological evaluation could have avoided 7 bronchoscopies and 1 lung biopsy (28).

## Discussion

Before the publication of the 2002 ATS guidelines, the diagnosis of ILD was based on histopathology. However, the interobserver agreement between expert histopathologists was reported low, especially in the presence of non-specific interstitial pneumonia (NSIP) pattern (43). The level of diagnostic accuracy and interobserver agreement between radiologists was better than between pathologists, and HRCT is currently the most used diagnostic tool in the evaluation of patients with ILD, being less invasive than lung biopsy. Furthermore, different histopathological findings may be present in different lobes of the same patient. Already before the publication of ATS/ERS/JRS/ALAT guidelines, the importance of a multidisciplinary evaluation of IPF patients was proposed (29). Current clinical practice guidelines suggest that in patients with suspected IPF a definite UIP pattern at HRCT could be considered a sufficient criterion for making the diagnosis. About half of the patients, however, presents a probable or inconsistent UIP pattern. In this group of patients the MDT is fundamental (44), especially for the identification of IPF which is the form of ILD with the worst prognosis with an average survival of 2–3 years from diagnosis. Given the current availability of effective anti-fibrotic drugs such as nintedanib and pirfenidone, a correct and early diagnosis of IPF is crucial (5).

SLB is generally considered in cases where imaging is inconsistent with UIP and in case of conflicting clinical data. Nevertheless, an UIP patter at histology is not necessarily indicative of IPF as demonstrated in the study by Tominaga, where the clinical information and HRCT images of 95 patients diagnosed as IPF and confirmed by a histological pattern compatible with UIP, were first re-evaluated separately and later on the course of MDD by a group of radiologists and pulmonologists. The two groups were progressively provided with more clinical data and radiological images. With the increase of clinical and radiological information, the degree of certainty in the diagnosis was reduced to a low or to an intermediate level in 41% of cases (37).

Multidisciplinary evaluation is essential in patients who do not have a definite UIP pattern at HRCT. Especially for probable UIP pattern, different studies have reported a variable frequency of IPF from 90 to 60%. Given the prognostic importance of a correct diagnosis, integration of imaging with clinical and histological data is fundamental, as demonstrated in a cohort of 179 patients with probable UIP pattern at HRCT in which the 50% of cases were diagnosed by MDD as IPF presenting worse prognosis compared to patients without IPF (27).

MDT classically include a pulmonologist, a radiologist and pathologist expert in ILD, but other professional figures including specialists in rheumatology, thoracic surgery, lung transplantation, and occupational medicine are often involved on demand (17). Despite the importance of MDD and available recommendations, there are no indications on the optimal composition of the MDT, on the timing or how to organize these meetings. Although in most cases the MDD aims to make an accurate diagnosis of ILD, the multidisciplinary approach can be used in patient care or for follow-up. Depending on the aims and degree of experience of the MDT itself, the organization may be different. For example, members of a recently established MDT could meet more frequently while in the case of clinicians with more experience in multidisciplinary discussion, the assessment could only be performed in selected cases. Depending on the purpose of the MDD, the members could be different, for example in the diagnostic evaluation the thoracic surgeon might not be useful (44).

Despite the recommendations and the available studies, it is currently not known whether the multidisciplinary approach is better than the single expert's clinical judgment in the diagnosis of patients with ILD. Moreover, the strict application of the guidelines for IPF is not always feasible; for example it is not always possible to perform SLB for safety reasons, and in the definition of the UIP pattern (both radiological and pathological) often the agreement between the observers is only moderate. Finally, the guidelines do not indicate how some clinical aspects, which may help to increase diagnostic confidence, should be included in MDD. This means that the multidisciplinary approach is not always applicable, and often the diagnosis is left to the opinion of the expert. The concept of “working diagnosis” recently proposed by the Fleischner Society allows to justify a disease-specific therapy despite a non-definite diagnosis (45). The lack of a standardized ontological framework can also determine heterogeneity in diagnosis for patients with ILD. Ryerson et al. made a proposal to standardize the terminology, by subdividing according to the degree of diagnostic confidence (> 90%, between 89 and 50% and <50%) the wording in the diagnosis of ILD in “confident,” “provisional,” and “unclassifiable ILD” (46). An international study involving 404 physicians that evaluated 60 cases of suspected IPF employed these standardized definitions to evaluate the impact of diagnostic likelihood on physician's decision to performed biopsy and on which treatment prescribe. This study showed that in presence of a provisional high confidence IPF diagnosis only a minority of patients (29.6%) would be addressed to SLB. Furthermore, most physicians prescribed anti-fibrotic therapy without performing histological evaluation in 63% of patients with a diagnostic likelihood of 70%, and in 63.0 and 41.5% of provisional high confidence and low confidence IPF diagnoses, respectively. The behavior of experts participating to this study was in most cases different from the guidelines; for instance, especially university hospital physicians tended not to require biopsy and to choose therapy according to a “working diagnosis” instead of a certain diagnosis as defined by the current guidelines. Therefore, the MDD would have a role in training physicians especially when they work in isolation (47).

The ATS guidelines emphasize the need to exclude the presence of a CTD during the evaluation of a patient with ILD. Despite this recommendation, rheumatologists are not considered mandatory among professional figures involved in the MDD, reserving the rheumatological evaluation only to patients with positive autoantibody serology, suspicious clinical manifestations for CTD and other rheumatological diseases, or in case with demographic characteristics atypical for IPF (e.g., female, age younger than 60 years, not smokers). The presence of a rheumatologist could therefore be fundamental in identifying specific non-pulmonary clinical manifestations that could not be easily recognized by traditional members of MDT, especially in patients with demographic, clinical and histopathological features inconsistent with IPF (15). For example, in female patients younger than 50 years, a diagnosis of IPF is unlikely compared to a male smoker over 60. Furthermore, some radiological patterns such as NSIP or organizing pneumonia (OP) are more characteristic of ILD associated with CTD. The presence of a definite UIP pattern, however, does not exclude the presence of an underlying autoimmune disease especially RA and some cases of SSc (48). Histological UIP pattern is indistinguishable between IPF and CTD-ILD, but some characteristics such as increased expression of lymphoid hyperplasia with germinal centers, more plasmatic infiltration, and less severe honeycombing are typical for CTD.

ILD can be a manifestation developed during an established CTD, so the diagnostic approach, therapy, and follow-up are better defined, and the rheumatologist is naturally involved in patient management. In other contexts, ILD may be the first manifestation at the onset of a not recognized CTD and the other typical clinical features may appear after the pulmonary involvement. This is known for example, especially in myositis spectrum disorders where in 10–30% of cases ILD may be the predominant manifestation (10), in particular in case of ASSD where the classical triad arthritis, myositis and ILD may develop during the follow up (49). The lack of specific classification criteria for ASSD makes the correct diagnosis for these patients more difficult, and an expert rheumatologist would be essential during the evaluation of these patients (12). Moreover, very few patients affected by SSc or RA may present as ILD at the onset, so in these cases the diagnostic process could be very challenging. In these pathological contexts the rheumatologist is crucial to identify the signs and symptoms more nuanced and less clear that cannot be recognized by other professional figures traditionally involved in the MDT.

The evaluation of the patient with ILD cannot be independent of the execution of blood tests, in particular autoimmunity, and different guidelines have proposed the execution of different biochemical test. The French guidelines recommends to evaluate complete blood cell count, CRP, serum creatinine, transaminases, γ-glutamyltransferase, and alkaline phosphatases, ANA, ACPA, and RF, reserving the search of other more specific antibodies (anti-SSA, anti-SSB, anti-centromeres, Scl70, anti-U3RNP, anti-synthetase antibodies, anti-thyroid antibodies) in case of positivity of first line antibodies or in presence of clinical manifestation compatible with CTD (50). The ATS/ERS/JRS/ALAT guideline recommends CRP, ESR, ANA (by immunofluorescence), RF, myositis panel and ACPA performing other test according to symptoms and signs (8). In the last few years, the diffusion of laboratory kits able to identify specific and associated myositis antibodies has made possible to reclassify patients with doubtful clinical pictures especially in the presence of negative ANA or with cytoplasmatic patterns. In particular antibodies such as MDA-5 and specific anti-synthetase antibodies such as PL2 and PL7 identify myositis with prevalent pulmonary expression that could be the first clinical manifestation up to 10–30% of cases of myositis spectrum disease (10).

The studies included in this review show that there is not a common behavior in serological evaluation, and only in 17 studies biochemical tests were evaluated during MDD. Fourteen studies reported the evaluation of autoantibodies without a clear suggestion of which test should be performed, and in 5 studies is not reported which serological test was chosen.

Another diagnostic challenge is represented by IPAF, a clinical entity of more recent characterization and of which classification criteria have been formulated (13). IPAF could be considered an ILD in which clinical or serological abnormalities typical of CTD are present but insufficient to satisfy classification criteria of a defined autoimmune disease. These classification criteria share many characteristics with undifferentiated connective tissues and allow to identify as IPAF very different clinical entities including patients with very early SSc or other CTD such as myositis spectrum diseases with a predominant pulmonary manifestation at onset. This could result in a mis-classification of patients especially without a rheumatologic evaluation (51).

Despite these considerations, no clear indications are available about the rheumatologist involvement in MDT. Only 7/29 studies included in this review described a rheumatological evaluation during MDD paying attention to the correct re-classification of patients who were initially classified as IPF (15, 16), and to the possibility of avoiding not necessary diagnostic procedures (28). From the available studies it is not possible to identify a univocal attitude on the modalities and timing of involvement of the rheumatologist in such a context.

**Figure 2:**
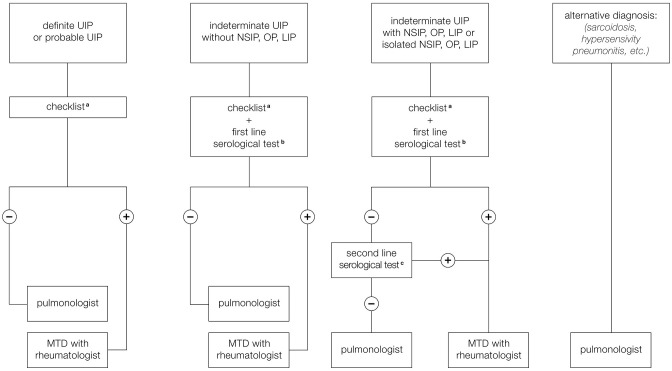
Proposal for a multidisciplinary team (MDT) involving the rheumatologist. (a) Checklist regarding signs and symptoms compatible with CTD or arthritis. (b) First line serological test: RF, ACPA, ANA, CPK. (c) Second line serological test: Anti-ds DNA, Anti-Ro (SS-A), Anti-La (SS-B), Anti-ribonucleoprotein, Anti-topoisomerase (Scl-70) Anti-tRNA synthetase, Anti-PM-Scl, Anti-MDA5.

For these reasons we have formulated a proposal for the organization of the MDT that provides different scenarios to suggest when and how the rheumatologist should be included in MDD, especially to help to identify CTD-ILD and IPAF (Figure 2). A first scenario includes ILD patients with HRCT pattern typical for UIP which is less frequent in cases of ILD associated with autoimmune diseases and more typical of IPF. However, it is still possible that a UIP pattern could be found, even if less frequently, in course of rheumatological disorders, especially RA and SSc. We have therefore proposed that the pulmonologist participating to MDT should be trained to identify clinical manifestations compatible with CTD or RA belonging to the checklist reported in Table 4. This core set includes main signs and symptoms typical of rheumatologic diseases that can be more frequently complicated with ILD: SSc, RA, Sjogren syndrome, and myositis spectrum disorder. For joint involvement, we have decided to include patients presenting at least one swollen or tender joint on examination excluding distal interphalangeal joints, first carpometacarpal joints, and first metatarsophalangeal in agreement with the definition reported in 2010 classification criteria for RA (54). For myositis spectrum disorders we have included the search for weakness of proximal musculature of the upper and lower limbs and for the presence of typical cutaneous manifestations (Gottron's papules and sign) described in the classification criteria of 2017 for idiopathic inflammatory myopathies (53). To identify patients affected by ASSD, fever, mechanic's hands, Raynaud's phenomenon and dysphagia have been included in the checklist. In particular, the last two manifestations together with puffy fingers, sclerodactyly, and telangiectasias, belong to scleroderma spectrum manifestations and so they should be considered as part of the coreset of clinical manifestations to be evaluated during diagnostic approach of patients with ILD. Finally, the sicca syndrome has been described according to the 2002 classification criteria for Sjogren's syndrome as a sensation of daily dryness, ocular or oral duration longer than 3 months (52). In case of positivity of at least one of these clinical criteria, we have proposed to involve the rheumatologist for a second evaluation in order to confirm the first clinical impression and therefore to perform further instrumental examinations, such as biochemical tests (including autoantibodies), capillaroscopy or echography suggested by the rheumatologist based on his clinical suspicion, thus avoiding useless and expensive investigations. Furthermore, this approach makes it possible to identify IPAF. According to the ATS classification criteria, in fact, being absent the morphological domain [HRCT pattern compatible with NSIP, OP or LIP (lymphoid interstitial pneumonia)], both the serological and the clinical domain are required. Therefore, our checklist including all the manifestations present in the clinical domain of these criteria, allows to identify patients with suspected IPAF and to confirm the suspicion after performing serological investigations.

**Table 4:** Signs and symptoms to be assessed in the suspicion of a rheumatological disease.

**Clinical manifestation of autoimmune disease**	**Description**
Joint involvement	Any swollen or tender joint on examination excluding distal interphalangeal joints, first carpometacarpal joints, and first metatarsophalangeal joints are excluded from assessment. Synovitis could be confirmed by imaging (Definition according 2010 Rheumatoid Arthritis Classification Criteria)
Raynaud syndrome	A vascular disorder especially of the fingers and toes, that is characterized by pallor, cyanosis, and redness in succession usually upon exposure to cold
Puffy fingers or sclerodactyly	Swelling or thickening of fingers
Distal digital tip ulceration	Loss of epithelialization and tissues involving, in different degrees, the epidermis, the dermis, the subcutaneous tissue, and sometimes also involving the bone
Telangiectasia	Small dilated-blood vessels near the surface of the skin or mucous membranes, measuring between 0.5 and 1 ml in diameter, especially localized on finger or face
Mechanics hand	Rough, cracked, hyperkeratotic, aspect of palmar areas of the fingers with fissures of the skin
Sicca syndrome	Sensation of dryness of eyes and/or mouth daily and persistent for 3 months (52)
Gottron signs	Fixed rash or patches on the extensor surfaces of the joints (especially elbows and/or knees)
Gottron papules	Erythematous to violaceous papules and plaques over the extensor surfaces of the metacarpophalangeal and interphalangeal joints
Eliotrophic rash	Violaceous erythema of the upper eyelids often with associated edema and telangiectasia
Fever	Unexplained by other causes
Muscle weakness	Weakness of proximal upper and lower extremities as Distal muscles are less involved. Weakness of neck flexors is usually more severe than of neck extensors (53)
Dysphagia	Difficulty in swallowing

Another scenario includes ILD patients with probable UIP pattern, indeterminate UIP pattern on HRCT. In this case, patterns frequently observed during CTD as NSIP, OP, and LIP would be included so the probability to observe an ILD secondary to an autoimmune disease is greater than in case of typical UIP pattern. For this reason, we have added to the clinical domain a biochemical screening test including ANA, RF, ACPA, and creatine phosphokinase (CPK). In case of negativity of both clinical and serological domain, patients presenting NSIP, OP, or LIP pattern are subjected to further serological evaluation in order to exclude IPAF or myositis spectrum disorders, and evaluated by a rheumatologist. In case of positivity of at least one of clinical or serological parameters during the screening, patients would be referred to rheumatologist that would suggest to perform further instrumental investigations such as biochemical tests (including autoantibodies), capillaroscopy, or echography based on clinical suspicion in order to avoid unnecessary investigations and to accurately diagnose patients with CTD-ILD (Figure 2).

## Conclusion

The role of the rheumatologist in MDD for the evaluation of patients with ILD is still not defined but could be fundamental for the correct diagnosis of CTD-ILD and IPAF. From the literature review, it emerges that in most cases the MDT is composed by the pulmonologist, radiologist and pathologist. The first being an essential member of the MDT, could be trained to be able to identify patients with suspected CTD-ILD and IPAF in order to select them for rheumatological evaluation. This organization could simplify the multidisciplinary meeting, reducing the times in which all professions are required for MDD. Our proposal for the organization of the MDT also provides a minimum core set of blood tests for screening, reserving the execution of second-level investigations only after a rheumatological indication and targeted according to the clinical suspicion, thus avoiding unnecessary and confounding tests.

## Author Contributions

FF and CS formulated the concept and design the paper. FF, GG, and AC performed SRL. FF wrote the manuscript. CS, GC, LC, and MG revised the manuscript critically, approved the final manuscript, and agreed to be accountable for all aspects of the manuscript.

### Conflict of Interest

The authors declare that the research was conducted in the absence of any commercial or financial relationships that could be construed as a potential conflict of interest.
